# Obesity disproportionately impacts lung volumes, airflow and exhaled nitric oxide in children

**DOI:** 10.1371/journal.pone.0174691

**Published:** 2017-04-04

**Authors:** Tsung-Chieh Yao, Hui-Ju Tsai, Su-Wei Chang, Ren-Hua Chung, Jing-Ya Hsu, Ming-Han Tsai, Sui-Ling Liao, Man-Chin Hua, Shen-Hao Lai, Li-Chen Chen, Kuo-Wei Yeh, Yu-Lun Tseng, Wan-Chen Lin, Su-Ching Chang, Jing-Long Huang, on behalf of the Prediction of Allergies in Taiwanese Children (PATCH) Study Group

**Affiliations:** 1 Division of Allergy, Asthma, and Rheumatology, Department of Pediatrics, Chang Gung Memorial Hospital and Chang Gung University College of Medicine, Taoyuan, Taiwan; 2 Chang Gung Immunology Consortium, Chang Gung Memorial Hospital and Chang Gung University College of Medicine, Taoyuan, Taiwan; 3 Community Medicine Research Center, Chang Gung Memorial Hospital at Keelung, Keelung, Taiwan; 4 Division of Biostatistics and Bioinformatics, Institutes of Population Health Sciences, National Health Research Institutes, Miaoli, Taiwan; 5 Department of Pediatrics, Feinberg School of Medicine, Northwestern University, Chicago, Illinois, United States of America; 6 Allergy and Clinical Immunology Research Center, National Cheng Kung University, Tainan, Taiwan; 7 Clinical Informatics and Medical Statistics Research Center, Chang Gung University College of Medicine, Taoyuan, Taiwan; 8 Department of Pediatrics, Chang Gung Memorial Hospital at Keelung, Keelung, Taiwan; 9 Division of Pediatric Pulmonology, Department of Pediatrics, Chang Gung Memorial Hospital, Taoyuan, Taiwan; Taipei City Hospital, TAIWAN

## Abstract

**Background:**

The current literature focusing on the effect of obesity and overweight on lung function and fraction of exhaled nitric oxide (FeNO) in children, particularly among healthy children of non-European descent, remains controversial. Furthermore, whether the relationship of obesity and overweight with lung function and FeNO in children is modified by atopy is unclear. The objective of this study was to examine the effect of excess weight on lung function parameters and FeNO among Asian children, with a particular focus on exploring the potential effect modification by atopy.

**Methods:**

We investigated the effect of excess weight on lung function and FeNO in a population sample of 1,717 children aged 5 to 18 years and explored the potential modifying effect of atopy.

**Results:**

There were positive associations of body mass index (BMI) z-score with forced vital capacity (FVC), forced expiratory volume in 1 second (FEV_1_), peak expiratory flow (PEF), and forced expiratory flow at 25–75% (FEF_25-75_) (all *P*<0.001), after controlling for confounders. The beta coefficient for FEV_1_ (0.084) was smaller than that for FVC (0.111). In contrast, a negative association was found between BMI z-score and FEV_1_/FVC ratio (*P*<0.001) and FeNO (*P* = 0.03). A consistent pattern of association for lung function variables was observed when stratifying by atopy. There was a negative association of BMI z-score with FeNO in atopic subjects (*P* = 0.006), but not in non-atopic subjects (*P* = 0.46).

**Conclusions:**

Excess weight disproportionately impacts lung volumes and airflow in children from the general population, independent of atopic status. Excess weight inversely affects FeNO in atopic but not in non-atopic children.

## Introduction

Excess weight represents a major global health challenge because of the established health risks and substantial increase in prevalence of overweight and/or obesity in children and adults worldwide, both in developed and developing countries [1–3]. In 2013, 23.8% of boys and 22.6% of girls were overweight or obese in developed countries [1]. Similar to many countries, Taiwan has experienced a substantial increase in the prevalence of childhood overweight and obesity, specifically, from 5.7% to 14.2% for overweight and from 7.9% to 17.4% for obesity in male students; and from 11.1% to 13.4% for overweight and from 3.1% to 4.1% for obesity in female students during the study period of 1991 and 2003 [3]. Excess weight is potentially associated with increased morbidity and mortality in children as well as in adults, which is attributed to obesity-related adverse health outcomes, including respiratory diseases (i.e., asthma, chronic obstructive pulmonary disease, and obstructive sleep apnea syndrome), metabolic syndrome, type 2 diabetes, cardiovascular diseases, and psychological problems [4]. One major concern is that excess weight in childhood is likely to persist into adulthood as revealed by several prospective studies [5]. During recent years, growing attention has therefore been directed towards the potential health consequences of childhood obesity and overweight.

Lung function, as assessed by the spirometric measures of forced expiratory volume in one second (FEV_1_), forced vital capacity (FVC), the FEV_1_ to FVC (FEV_1_/FVC) ratio, peak expiratory flow (PEF), and forced expiratory flow at 25–75% (FEF_25-75_), is not only an objective indicator of general respiratory health but also an important long-term predictor of all-cause mortality and morbidity in adults [6–10]. The fraction of exhaled nitric oxide (FeNO) is a non-invasive biomarker of eosinophilic airway inflammation, which has emerged in recent years to aid in the diagnosis and management of respiratory diseases in clinical practice [11]. Atopy has been reported to be an important determinant of FeNO, even in healthy subjects who have no current or past symptoms suggestive of allergic diseases [12]. Obesity has been found to be associated with reduced lung function and FeNO in adult subjects [13–16]. Yet, the current literature focusing on the effect of obesity and overweight on lung function and FeNO in children, particularly among healthy children of non-European descent, remains controversial. Furthermore, whether the relationship of obesity and/or overweight with lung function and FeNO in children is modified by atopy is largely unknown. Our study objective was to examine the effect of excess weight on lung function parameters and FeNO among Asian children aged 5 to 18 years in a well characterized population-based study, with a particular focus on exploring the potential effect modification by atopy.

## Methods

### Study subjects

**Fig 1:**
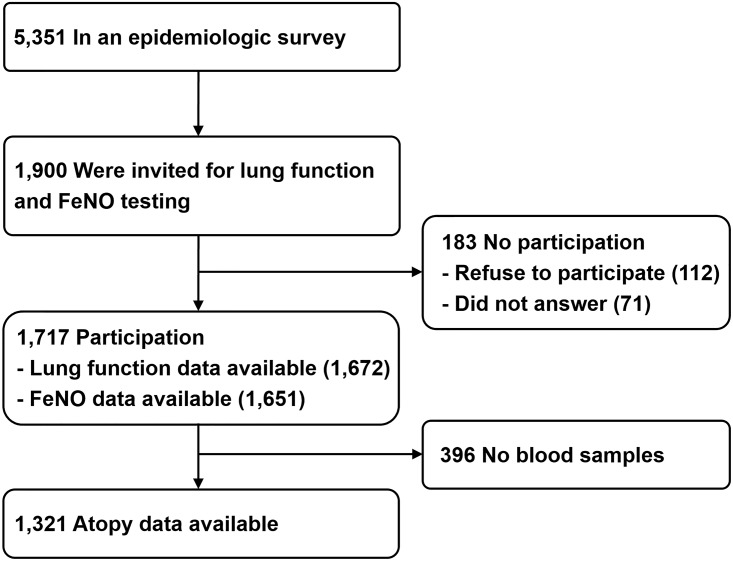
Schematic presentation of the recruitment process of the study subjects.

This study included 1,717 subjects participating in the Prediction of Allergies in Taiwanese CHildren (PATCH) study, a population-based prospective cohort study that was launched in 2007 in Taiwan. In this study, we used a modified ISAAC questionnaire for collecting epidemiologic and clinical data. Subject recruitment and data collection for the PATCH study have been described elsewhere [11, 12, 17–22]. The flow diagram for subject recruitment of the current study is shown in Fig 1. In brief, among the 5,351 subjects who participated in an epidemiologic survey, a random sample of 1,900 schoolchildren aged 5 to 18 years were invited to undergo lung function and FeNO measurements unless the subjects’ underlying diseases preclude them from testing. A total of 1,717 children (90.4%) participated in the lung function and FeNO tests in December 2007 through January 2009. Parents of all study participants answered a questionnaire regarding demographic data, general health information, and questions related to clinical symptoms and diagnosis of allergic diseases. All subjects were born to parents who were Asian descent. The Institutional Review Board of Chang Gung Medical Foundation approved the study (96-0370B and 103-6485A3). All experiments in this study were performed in accordance with the relevant guidelines and regulations. Written informed consent was obtained from the parents of all subjects on behalf of their children enrolled in this study. Verbal assent was also obtained from children over 7 years of age.

### Anthropometric measurements

All participants had their weights and heights measured according to a standard protocol. Weight (in kg) was measured to the nearest 0.1 kg and height (in cm) was also measured to one decimal. Body mass index (BMI) was defined as weight (kg) divided by the height squared (m^2^): BMI = kg/m^2^. Age- and sex-specific BMI z-scores were calculated by the lambda-mu-sigma (LMS) method using the following formula:

$$\text{z}=\left({\left(\text{BMI}/\text{M}\right)}^{\text{L}}-1\right)/\left(\text{L}\times \text{S}\right),$$

where the values for L (skewness λ), M (median μ), and S (coefficient of variation σ) correspond to skewness, median, and coefficient of variation as quantities that change smoothly with child's age that were derived from an international survey of six large nationally representative cross-sectional growth studies [23–25]. For the categorical analyses, BMI category was also determined by using a set of age- and sex-specific BMI cut-off values for ages 2–18 years proposed by the International Obesity Task Force (IOTF) that were constructed to pass the adult BMI cut-offs at age 18 for thinness (<17 kg/m^2^), normal weight (≧17 and <25 kg/m^2^), overweight (≧25 and <30 kg/m^2^), and obesity (≧30 kg/m^2^) [23–25].

### Lung function measurements

Lung function was measured using spirometry (Spirolab II, Medical International Research, Roma, Italy) in accordance with the American Thoracic Society (ATS)/ European Respiratory Society (ERS) recommendations [26]. Three technically acceptable forced expirations were performed for up to 8 tests. The highest FVC, FEV_1_, PEF, and FEF_25-75_ were recorded and used in the subsequent analyses.

### FeNO

FeNO measurements were performed by a chemiluminescence analyzer (CLD 88sp NO analyzer^®^, Eco Medics, Duernten, Switzerland) according to the 2005 ATS/ERS recommendations for standardized online measurement, as described elsewhere [11, 12].

### Atopy/Asthma

Allergen-specific serum IgE was measured by ImmunoCAP^™^ Phadiatop^®^ Infant (Phadia, Uppsala, Sweden), a reliable alternative to skin prick tests for detecting allergic sensitization [27]. The following allergens were included: house dust mite, cat, dog, birch, timothy, ragweed, wall pellitory (a local plant), egg white, cow’s milk, peanut, and shrimp. Atopy (also known as allergic sensitization) was defined as a positive Phadiatop Infant test result (≥0.35 PAU/L). In addition, asthmatic children were defined as those having physician-diagnosed asthma.

### Statistical analysis

All data analyses were performed using the SPSS statistical package version 15.0 for Windows (SPSS, Chicago, IL, USA). Subjects were categorized into four groups: thinness, normal weight, overweight, and obesity based on age- and sex-specific BMI cut-offs, as described above. Categorical variables were compared using chi-squared test or Fisher's exact test, as appropriate. Comparisons of lung function parameters and FeNO across BMI categories were analyzed using analysis of covariance (ANCOVA). Multivariable linear regression was used to examine the relationship of BMI categories with lung function parameters and FeNO. The adjusted covariates included age, sex, physician-diagnosed asthma, and smoking status. We also treated BMI z-score as a continuous variable in the analyses. Linear trends were tested for significance by regressing lung function measures and FeNO, separately, on BMI z-score, with adjustment for the covariates listed above. We performed subgroup analyses stratified by atopy to evaluate potential effect modifications. To obtain approximate normality, FeNO values were logarithmically transformed before the analysis. A *P*-value <0.05 was considered statistically significant.

## Results

### Subject characteristics

The demographic and clinical characteristics of the 1,717 subjects are listed in Table 1. The mean age and corresponding standard deviation (SD) were 10.3 years (SD: 2.6 years [range 5–18]) and 49% of subjects were male. The prevalence of thinness, normal weight, overweight, and obesity among the whole study population was 2.0%, 70.0%, 21.1%, and 6.9%, respectively. In total, 10.6% of the study population had been diagnosed by a physician as having asthma at some time in the past. The median FeNO and corresponding interquartile range (IQR) were 16.3 ppb (10–34.7) in total study sample; 28.3 ppb (14.3–49) for children with atopy; 11.3 ppb (8–16.3) for children without atopy; 32.2 ppb (13.6–55.2) for children with physician-diagnosed asthma; 16 ppb (9.7–31) for children without physician-diagnosed asthma. There was no notable difference in terms of age, sex, and prevalence of physician-diagnosed asthma between these 1,717 participants and the original 5,351 cohort members (*P* = 0.1 for age; *P* = 0.98 for sex; and 10.6% of physician-diagnosed asthma in the study sample of 1,717 participants vs. 9.8% of physician-diagnosed asthma in the original 5,351 cohort members). Acceptable lung function and FeNO measurements were obtained in 1,672 (97.4%) and 1,651 (96.2%) of 1,717 study subjects. Atopy data was available in 1,321 of 1,717 (76.9%) subjects who provided blood samples for testing.

**Table 1:** Demographic and clinical characteristics of study subjects.

Characteristic	Sample size	Data
**Age (year) (mean ± SD)**	1,717	10.3 ± 2.6
**Sex [n (%)]**	1,717	
**Male**		842 (49.0)
**Female**		875 (51.0)
**Anthropometric measurement (mean ± SD)**	1,717	
**Weight (kg)**		37.1 ± 13.3
**Height (cm)**		138.7 ± 14.7
**BMI (kg/m**^**2**^**)**		18.7 ± 3.6
**BSA (m**^**2**^**)**		1.19 ± 0.26
BMI category [n (%)]*	1,717	
**Thinness**		33 (2.0)
**Normal weight**		1,201 (70.0)
**Overweight**		364 (21.1)
**Obesity**		119 (6.9)
**Lung function (mean ± SD)**	1,672	
**FVC (L)**		2.08 ± 0.66
**FEV**_**1**_ **(L)**		1.80 ± 0.57
**FEV**_**1**_**/FVC ratio (%)**		86.84 ± 6.10
**PEF (L/s)**		3.46 ± 1.11
**FEF**_**25-75**_ **(L/s)**		2.18 ± 0.78
**FeNO (ppb) [median (IQR)]**	1,651	16.3 (10, 34.7)
**Physician-diagnosed asthma [n (%)]**	1,688	179 (10.6)
**Atopy**	1,321	757 (57.3)
**Active Smoking [n (%)]**	1,717	10 (0.6)
**Household passive smoking [n (%)]**	1,657	870 (52.5)

BMI: body mass index; BSA: body surface area; FVC: forced vital capacity; FEV_1_: forced expiratory volume in 1 second; PEF: peak expiratory flow; FEF_25-75_: forced expiratory flow at 25–75%; FeNO: fraction of exhaled nitric oxide; ppb, parts per billion; IQR: interquartile range.

* BMI categories were determined by using a set of age and sex specific BMI cut-off values for ages 2–18 years proposed by the International Obesity Task Force that were constructed to pass the adult BMI cut-offs at age 18 for thinness (<17 kg/m^2^), normal weight (≧17 and <25 kg/m^2^), overweight (≧25 and <30 kg/m^2^) and obesity (≧30 kg/m^2^).

Table 2 presents the prevalence of BMI categories stratified by sex, age, and physician-diagnosed asthma, respectively. The prevalence of obesity and overweight in boys (10.0% and 23.8%, respectively) was significantly higher than that in girls (4.0% and 18.7%, respectively) (*P* <0.001 for obesity and *P* = 0.01 for overweight, respectively) (Table 2). The prevalence of obesity in younger children aged 5–7 years (8.8%) and 8–9 years (7.8%) was significantly higher than in adolescents aged 13–18 years (2.8%) (*P* = 0.003 for children aged 5–7 years versus adolescents aged 13–18 years; and *P* = 0.007 for children aged 8–9 years versus adolescents aged 13–18 years, respectively) (Table 2). A higher prevalence of obesity was observed in children with asthma (10.6%) as compared to those without asthma (6.5%) (*P* = 0.03) (Table 2).

**Table 2:** Association of BMI categories with sex, age and asthma, respectively*.

Variable	*n*	BMI category				*P*
		Thinness	Normal weight	Overweight	Obesity	
**Sex**						
Male	842 (49.0)	11 (1.3)	547 (65.0)	200 (23.8)	84 (10.0)	**<0.001**
Female	875 (51.0)	22 (2.5)	654 (74.7)	164 (18.7)	35 (4.0)	
**Age (years)**						
5–7	388 (26.6)	7 (1.8)	271 (69.8)	76 (19.6)	34 (8.8)	**0.02**
8–9	461 (26.8)	5 (1.1)	316 (68.5)	104 (22.6)	36 (7.8)	
10–12	618 (36.0)	11 (1.8)	427 (69.1)	138(22.3)	42 (6.8)	
13–18	250 (14.6)	10 (4.0)	187 (74.8)	46 (18.4)	7 (2.8)	
**Asthma**						
Yes	179 (10.6)	0 (0)	117 (65.4)	43 (24.0)	19 (10.6)	**0.03**
No	1,509 (89.4)	33 (2.2)	1,066 (70.6)	312 (20.7)	98 (6.5)	

BMI: body mass index.

* BMI categories were determined by using a set of age and sex specific BMI cut-off values for ages 2–18 years proposed by the International Obesity Task Force that were constructed to pass the adult BMI cut-offs at age 18 for thinness (<17 kg/m^2^), normal weight (≧17 and <25 kg/m^2^), overweight (≧25 and <30 kg/m^2^) and obesity (≧30 kg/m^2^).

### Relationship of BMI category with lung function and FeNO

**Fig 2:**
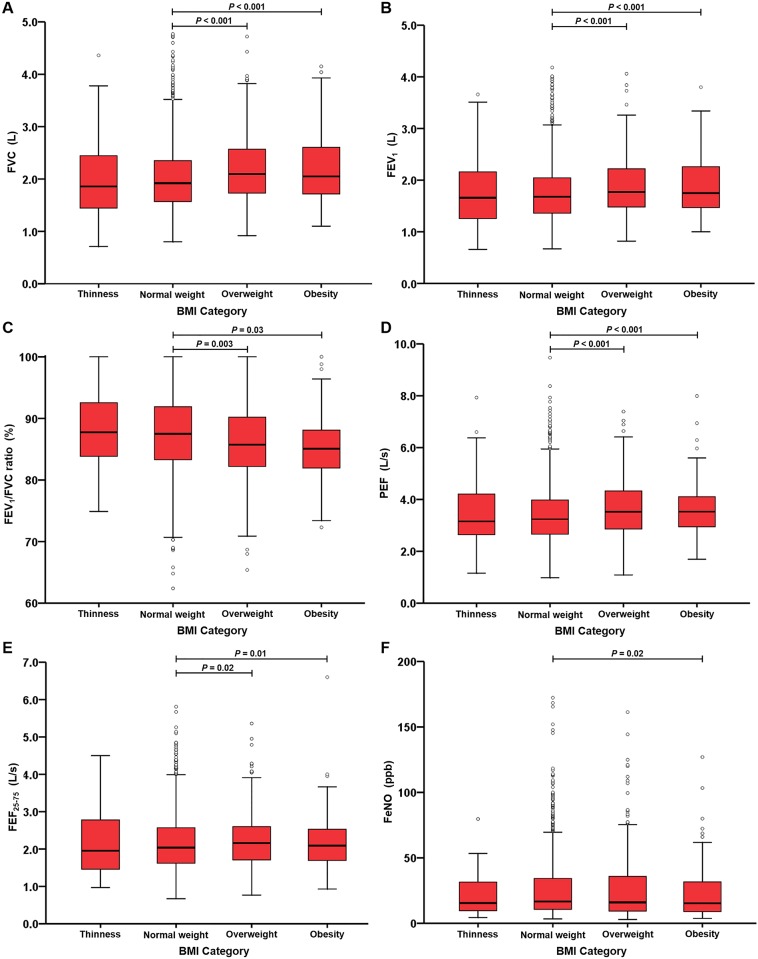
Box plots showing median and interquartile ranges of lung function parameters by BMI category. (A) FVC and (B) FEV_1_, (C) FEV_1_/FVC ratio, (D) PEF, (E) FEF_25-75_, (F) FeNO. *P* values refer to the comparisons using the ANCOVA model.

FVC, FEV_1_, FEV_1_/FVC ratio, PEF, FEF_25-75_, and FeNO in subjects grouped by 4 different BMI categories are shown in Fig 2. Obese (2.21 ± 0.06 L [mean ± SE]) and overweight (2.20 ± 0.03 L) subjects both had a significantly higher FVC (both *P* <0.001) compared to normal-weight subjects (2.04 ± 0.02 L), while the findings in the underweight (2.0 ± 0.15 L) and normal-weight groups were similar (Fig 2). Likewise, obese and overweight subjects both had a significantly higher FEV_1_, PEF, and FEF_25-75_ compared to normal-weight subjects (all *P* <0.05). Obese (85.15 ± 0.50% [mean ± SE]) and overweight (85.82 ± 0.32%) subjects had a significantly lower FEV_1_/FVC ratio (*P* = 0.03 and *P* = 0.003, respectively) compared to normal-weight subjects (87.29 ± 0.18%) (Fig 2). Obese subjects had a lower FeNO level (median: 15.3 p.p.b; interquartile range (IQR): 9–31.7) compared to normal-weight subjects (median: 16.7 p.p.b; IQR: 10.7–34.3) (*P* = 0.02).

Multivariable regression analysis was undertaken to explore the relationship of BMI categories with lung function, adjusting for age, sex, physician-diagnosed asthma and active smoking. After adjusting for the above covariates, BMI categories were significantly associated with all lung function parameters including FVC, FEV_1_, FEV_1_/FVC ratio, PEF, and FEF_25-75_ (Table 3). Specifically, obesity and overweight were positively and significantly associated with FVC, with a mean difference of 229 mL (for obesity; *P* <0.001) and 144 mL (for overweight; *P* <0.001) in FVC between the subjects with obesity and overweight, respectively, and those with normal weight. Likewise, obesity and overweight were positively and significantly associated with FEV_1_, with a mean difference of 171 mL (for obesity; *P* <0.001) and 101 mL (for overweight; *P* <0.001) in FEV_1_ between the subjects with obesity and overweight, respectively, and those with normal weight. In contrast, obesity (*P* = 0.04) and overweight (*P* = 0.003) were negatively significantly associated with FEV_1_/FVC ratio, respectively. The mean difference in FEV_1_/FVC ratio between subjects with obesity and those with normal weight was 1.25% and the mean difference was 1.12% between subjects with overweight and those with normal weight. Similar results were found when subjects with asthma were excluded from the analyses (Table 3). Of note, Lee *et al*. reported that pulmonary function indices could be influenced by different age, sex, height, and weight, but they only examined three pulmonary function indices in their previous work [28]. Instead of using raw data for FVC, FEV_1_, FEV_1_/FVC, PEF and FEF_25-75_, we have used percentage of predicted values for FVC, FEV_1_, FEV_1_/FVC, PEF and FEF_25-75_; and repeated the analyses to examine the association between obesity and pulmonary function. Similar results were observed when comparing with those using raw data for FVC, FEV_1_, FEV_1_/FVC, PEF and FEF_25-75_ (S1–S3 Tables).

**Table 3:** Multivariable analysis of associations of BMI categories with lung function variables and FeNO*.

	Total subjects (*n* = 1,717)^$^		Subjects without asthma (*n* = 1,509)^†^	
	β (95% CI)	*P*	β (95% CI)	*P*
**FVC (L)**				
Thinness	-0.113 (-0.243, 0.016)	0.09	-0.112 (-0.243, 0.019)	0.09
Normal weight	Reference	-	Reference	-
Overweight	0.144 (0.1, 0.188)	**<0.001**	0.146 (0.098, 0.194)	**<0.001**
Obesity	0.229 (0.159, 0.300)	**<0.001**	0.244 (0.166, 0.322)	**<0.001**
**FEV**_**1**_ **(L)**				
Thinness	-0.091 (-0.202, 0.019)	0.10	-0.091 (-0.203, 0.02)	0.11
Normal weight	Reference	-	Reference	-
Overweight	0.101 (0.063, 0.138)	**<0.001**	0.096 (0.055, 0.137)	**<0.001**
Obesity	0.171 (0.111, 0.231)	**<0.001**	0.188 (0.121, 0.254)	**<0.001**
**FEV**_**1**_**/FVC ratio (%)**				
Thinness	0.341 (-1.789, 2.472)	0.75	0.298 (-1.801, 2.398)	0.78
Normal weight	Reference	-	Reference	-
Overweight	-1.118 (-1.849, -0.387)	**0.003**	-1.392 (-2.159, -0.624)	**<0.001**
Obesity	-1.249 (-2.416, -0.082)	**0.04**	-1.181 (-2.43, 0.068)	0.06
**PEF (L/s)**				
Thinness	-0.112 (-0.373, 0.150)	0.40	-0.115 (-0.376, 0.146)	0.39
Normal weight	Reference	-	Reference	-
Overweight	0.187 (0.097, 0.276)	**<0.001**	0.157 (0.061, 0.252)	**0.001**
Obesity	0.299 (0.156, 0.422)	**<0.001**	0.312 (0.157, 0.468)	**<0.001**
**FEF**_**25-75**_ **(L/s)**				
Thinness	-0.116 (-0.306, 0.075)	0.23	-0.12 (-0.311, 0.071)	0.22
Normal weight	Reference	-	Reference	-
Overweight	0.075 (0.009, 0.14)	**0.03**	0.052 (-0.018, 0.122)	0.14
Obesity	0.139 (0.035, 0.243)	**0.009**	0.163 (0.05, 0.277)	**0.005**
**Ln FeNO (ppb)**				
Thinness	-0.094 (-0.367, 0.18)	0.50	-0.094 (-0.364, 0.176)	0.49
Normal weight	Reference	-	Reference	-
Overweight	-0.072 (-0.168, 0.024)	0.14	-0.068 (-0.168, 0.033)	0.19
Obesity	-0.176 (-0.332, -0.021)	**0.03**	-0.212 (-0.379, -0.046)	**0.01**

BMI: body mass index; FeNO: fraction of exhaled nitric oxide; CI: confidence interval; FVC: forced vital capacity; FEV_1_: forced expiratory volume in 1 second; PEF: peak expiratory flow; FEF_25-75_: forced expiratory flow at 25–75%; ppb, parts per billion.

* BMI categories were determined by using a set of age and sex specific BMI cut-off values for ages 2–18 years proposed by the International Obesity Task Force that were constructed to pass the adult BMI cut-offs at age 18 for thinness (<17 kg/m^2^), normal weight (≧17 and <25 kg/m^2^), overweight (≧25 and <30 kg/m^2^) and obesity (≧30 kg/m^2^). *P* values less than 0.05 are in bold.

^$^Among 1,717 study subjects acceptable lung function and FeNO measurements were obtained in 1,672 and 1,651 subjects, respectively. Adjusted for age, sex, asthma, and active smoking.

^†^208 subjects with asthma (n = 179) or missing data (n = 29) were excluded from the analyses. Adjusted for age, sex, and active smoking.

### Relationship of BMI z-score with lung function and FeNO

We also explored the relationship between BMI z-scores (as a continuous variable) and lung function variables as well as FeNO (Table 4). After controlling for covariates, there were significant positive associations of BMI z-score with FVC, FEV_1_, PEF, and FEF_25-75_, individually (all *P* <0.001) (Table 4). In contrast, a significant negative association of BMI z-score with FEV_1_/FVC ratio (*P* <0.001) and FeNO (*P* = 0.03) was found (Table 4). Of note, the beta coefficient for FEV_1_ (0.084) was relatively lower with respect to that for FVC (0.111), indicating a disproportionate increase in FEV_1_ as compared to the corresponding increase in FVC with increasing BMI z-score. The aforementioned associations remained significant after excluding subjects with asthma from the analyses (Table 4). Furthermore, similar results were observed when we examined subjects with asthma (S4 Table).

**Table 4:** Multivariable analysis of associations of BMI z-scores with lung function variables and FeNO*.

	Total subjects (*n* = 1,717)^$^		Subjects without asthma (*n* = 1,509)^†^	
	β (95% CI)	*P*	β (95% CI)	*P*
**FVC (L)**	0.111 (0.096, 0.126)	**<0.001**	0.115 (0.099, 0.131)	**<0.001**
**FEV**_**1**_ **(L)**	0.084 (0.071, 0.097)	**<0.001**	0.088 (0.074, 0.101)	**<0.001**
**FEV**_**1**_**/FVC ratio (%)**	-0.598 (-0.854, -0.342)	**<0.001**	-0.606 (-0.873, -0.339)	**<0.001**
**PEF (L/s)**	0.141 (0.11, 0.172)	**<0.001**	0.135 (0.102, 0.168)	**<0.001**
**FEF**_**25-75**_ **(L/s)**	0.069 (0.046, 0.091)	**<0.001**	0.073 (0.049, 0.097)	**<0.001**
**Ln FeNO (ppb)**	-0.037 (-0.071, -0.004)	**0.03**	-0.04 (-0.075, -0.005)	**0.03**

BMI: body mass index; FeNO: fraction of exhaled nitric oxide; CI: confidence interval; FVC: forced vital capacity; FEV_1_: forced expiratory volume in 1 second; PEF: peak expiratory flow; FEF_25-75_: forced expiratory flow at 25–75%; ppb, parts per billion.

* BMI z-score was treated as a continuous variable. *P* values less than 0.05 are in bold.

^$^Among 1,717 study subjects acceptable lung function and FeNO measurements were obtained in 1,672 and 1,651 subjects, respectively. Adjusted for age, sex, asthma, and active smoking.

^†^ 208 subjects with asthma (n = 179) or missing data (n = 29) were excluded from the analyses. Adjusted for age, sex, and active smoking.

On the other hand, a significant negative association of BMI z-score with FeNO (*P* = 0.03) was found (Table 4). Furthermore, we also added FEV_1_/FVC ratio as a covariate in the analysis to address whether airway obstruction accounts for the negative association of BMI z-score with FeNO, which yielded a significant negative relationship between BMI z-score and FeNO (*P* = 0.03).

### Effect modification by atopy

**Fig 3:**
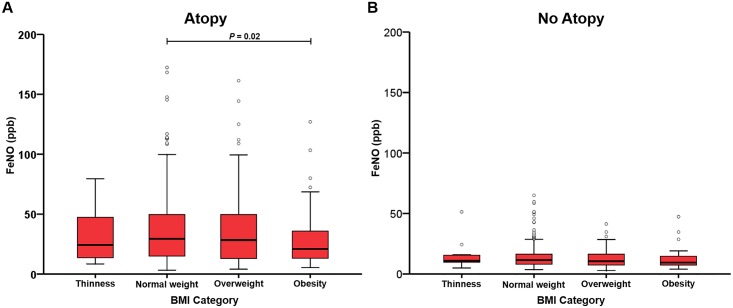
Box plots showing median and interquartile ranges of FeNO by BMI category, stratified by atopy. (A) Atopy, (B) No atopy. *P* values refer to the comparisons using the ANCOVA model.

We then performed stratified analysis to evaluate whether the effects of excess weight on lung function and FeNO is modified by atopy (Table 5). Regarding lung function variables, stratified analyses showed a consistent pattern of findings with small changes in the magnitude of the beta coefficients in atopic and non-atopic subjects, respectively (Table 5). In contrast, there was a negative association of BMI z-score with FeNO in atopic subjects (*P* = 0.006), but such association was not observed in non-atopic subjects (*P* = 0.46) (Table 5). As presented in Fig 3, FeNO levels in obese subjects were significantly lower than that in normal-weight subjects in atopic group (*P* = 0.02), while no such difference was seen in non-atopic group. We further examined potential interactive effect of BMI z-scores and atopy on lung function parameters and FeNO. We found borderline significant interaction between BMI z-scores and atopy on FVC (*P* = 0.08) and FEV_1_ (*P* = 0.07). We also used total serum IgE to define atopy at a customary cutoff of 100 kU/L and a previously reported optimal cutoff of 77.7 kU/L [19] as a sensitivity test, which yielded similar results (Table 5).

**Table 5:** Multivariable analysis of associations of BMI z-scores with lung function variables and FeNO, stratified by atopy*.

	Atopy (*n* = 757)^$^		No Atopy (*n* = 564)^$^	
	β (95% CI)	*P*	β (95% CI)	*P*
**FVC (L)**	0.101 (0.078, 0.124)	**<0.001**	0.133 (0.105, 0.161)	**<0.001**
**FEV**_**1**_ **(L)**	0.075 (0.055, 0.095)	**<0.001**	0.103 (0.081, 0.125)	**<0.001**
**FEV**_**1**_**/FVC ratio (%)**	-0.505 (-0.899, -0.112)	**0.01**	-0.711 (-1.148, -0.274)	**0.001**
**PEF (L/s)**	0.139 (0.093, 0.186)	**<0.001**	0.171 (0.114, 0.228)	**<0.001**
**FEF**_**25-75**_ **(L/s)**	0.061 (0.026, 0.097)	**0.001**	0.083 (0.045, 0.12)	**<0.001**
**Ln FeNO (ppb)**	-0.072 (-0.124, -0.021)	**0.006**	-0.015 (-0.055, 0.025)	0.46
	**IgE > = 100 kU/L (*n* = 615)**		**IgE< 100 kU/L (*n* = 706)**	
**FVC (L)**	0.107 (0.081, 0.134)	**<0.001**	0.122 (0.098, 0.146)	**<0.001**
**FEV1 (L)**	0.08 (0.056, 0.103)	**<0.001**	0.094 (0.074, 0.113)	**<0.001**
**FEV1/FVC ratio (%)**	-0.574 (-1.011, -0.137)	**0.01**	-0.613 (-1.008, -0.218)	**0.002**
**PEF (L/s)**	0.146 (0.094, 0.199)	**<0.001**	0.161 (0.112, 0.211)	**<0.001**
**FEF25-75 (L/s)**	0.06 (0.021, 0.099)	**0.003**	0.081 (0.047, 0.115)	**<0.001**
**Ln FeNO (ppb)**	-0.071 (-0.128, -0.014)	**0.02**	-0.021 (-0.061, 0.018)	0.29
	**IgE > = 77.7 kU/L (*n* = 696)**		**IgE < 77.7 kU/L (*n* = 625)**	
**FVC (L)**	0.114 (0.089, 0.139)	**<0.001**	0.116 (0.09, 0.141)	**<0.001**
**FEV1 (L)**	0.085 (0.064, 0.107)	**<0.001**	0.089 (0.068, 0.109)	**<0.001**
**FEV1/FVC ratio (%)**	-0.62 (-1.028, -0.212)	**0.003**	-0.547 (-0.968, -0.126)	**0.01**
**PEF (L/s)**	0.15 (0.1, 0.199)	**<0.001**	0.157 (0.104, 0.209)	**<0.001**
**FEF25-75 (L/s)**	0.067 (0.030, 0.102)	**<0.001**	0.076 (0.039, 0.113)	**<0.001**
**Ln FeNO (ppb)**	-0.066 (-0.121, -0.011)	**0.02**	-0.016 (-0.056, 0.024)	0.44

BMI: body mass index; FeNO: fraction of exhaled nitric oxide; CI: confidence interval; FVC: forced vital capacity; FEV_1_: forced expiratory volume in 1 second; PEF: peak expiratory flow; FEF_25-75_: forced expiratory flow at 25–75%; ppb, parts per billion; IgE: Immunoglobulin E.

* BMI z-score was treated as a continuous variable. Atopy was defined as a positive Phadiatop Infant test (≥0.35 PAU/L). We also used total serum IgE to define atopy at a customary cutoff of 100 kU/L and a previously reported optimal cutoff of 77.7 kU/L [19] as a sensitivity test. Adjusted for age, sex, asthma, and active smoking. *P* values less than 0.05 are in bold.

^$^ Atopy data was available in 1,321 of 1,717 subjects who provided blood samples for testing. As a result, number of subjects evaluated in Table 5 is a subset of subjects examined in Tables 3 and 4.

### Effect of active smoking and influence of airway obstruction

Furthermore, to examine the potential effect of active smoking, we excluded subjects with active smoking from the study sample and repeated all the analyses. Similar results were found as those presented in Tables 2–5 after excluding children with active smoking from the study sample. In addition, we have further treated FEV_1_/FVC as a covariate to control the influence of airway obstruction on FeNO. Similar results as those presented in Tables 4 & 5 were found, no matter with or without adjusting FEV_1_/FVC in the models.

## Discussion

Obesity is a worldwide public health problem that poses a significant health threat as well as a considerable economic and societal burden. This study investigated the relationship of BMI status with lung function and FeNO in a population-based study of 1,717 Asian children. The major finding of the current study is that increasing BMI disproportionately impacts lung volumes and airflow among children in a population setting which is reflected by increase in FVC, FEV_1_, and PEF, and FEF_25-75_, but decrease in FEV_1_/FVC ratio, independent of atopic status. Another interesting finding is that increasing BMI is inversely associated with FeNO in atopic but not in non-atopic children. To our knowledge, this is one of the first population-based studies to investigate the relationship between obesity, lung function, and FeNO in a large population sample of Asian children according to atopic status.

Breathlessness on exertion is a very common symptom among obese subjects [29]. Obesity has been shown to adversely affect lung function in adults [13–15]. Previous studies have shown that in adults, increasing BMI is associated with a restrictive ventilatory deficit, with lower FEV_1_ and FVC, but relatively preserved FEV_1_/FVC ratio [13, 14]. In contrast to the above findings in adults, increasing BMI is significantly associated with an increase in FVC, FEV_1_, PEF, and FEF_25-75_, but a decrease in FEV_1_/FVC ratio in a large population sample of 1,717 Asian children in the current study. We found that FVC, a measure of lung volume, increased linearly with increasing BMI; on the other hand, FEV_1_ and PEF, measures of airflow limitation, also increased linearly with increasing BMI. Although increasing BMI is associated with increases in both FVC and FEV_1_, the increase in FEV_1_ is relatively smaller that is approximately three-fourths of the corresponding increase in FVC, thereby decreasing the FEV_1_/FVC ratio which reflects a reduction in airflow. These findings could be attributed to disproportional growth of airways relative to lung parenchyma (a concept called dysanapsis), whereby lung size is larger in obese children than in normal-weight children but airway size has not yet grown proportionately [30]. This phenomenon might partly responsible to the reported association between obesity and asthma in our recent work as well as other studies [18].

There have been few studies assessing the relationship of obesity with lung function across different study populations of healthy children [31–34]. In accordance with our results, Han et al. have demonstrated that increasing BMI is associated with higher FVC and FEV_1_, and lower FEV_1_/FVC ratio among a population sample of U.S. children without asthma [31]. Similarly, Cibella et al. have shown that weight is positively correlated to FVC and FEV_1_ but negatively correlated to FEV_1_/FVC ratio in adolescents in an Italy study [32]. Similar findings have been reported in children with asthma [35]. Other studies have either failed to find a link between indicators of obesity and lung function in children or have reported inconsistent results [33, 34, 36]. Nonetheless, it remains unclear whether the effect of obesity on lung function is consistent between atopic and non-atopic children. To our knowledge, this is one of the first studies attempting to examine indicators of excess weight and lung function in children according to their atopic status. When stratifying by atopic status, we demonstrate that increasing BMI is significantly associated with lung function in both atopic and non-atopic children.

One interesting finding of the current study is that increasing BMI is negatively associated with FeNO levels in atopic children, but not in non-atopic children. FeNO is established as a robust, non-invasive biomarker of allergic airway inflammation [11, 12]. A previous study reported by our group has demonstrated a previously unrecognized negative independent association between BMI and FeNO levels in children [11]. The finding from this study has extended our understanding of the relationship between excess weight and FeNO, showing that the association is mainly confined to atopic subjects. Based on the existing literature, limited studies have provided suggestive evidence on this issue and underlying biological mechanisms have remained largely unknown. In addition, even we included 757 study subjects with atopy and 564 study subjects without atopy in the present study, it was likely we might encounter insufficient sample size and lack of analytical power to detect the impact of atopy on the correlation between FeNO and BMI in non-atopic subjects. Therefore, further investigation on this issue would be merited. Even though the exact mechanisms remain unknown, it is of clinical importance to recognize this association when FeNO is clinically evaluated in children, particularly if they are obese and/or atopic.

Ethnic differences of FeNO levels between White and Asian children have been reported in our earlier study and previous Western studies, as FeNO levels are significantly higher in Asian children than children of European descent [12, 37–39]. Potential explanations for the observed higher levels of FeNO in children in the current study than in previous studies include ethnic differences in the genetic regulation of nitric oxide synthase pathways, different methodological factors, various measurement conditions, heterogeneity of population, and environmental factors such as air pollution, type of diets, differences in oral bacterial flora, and respiratory infections etc. [12].

This study has considerable strengths, including a large population sample of children across a broad age range recruited from community, a high participation rate, and performance of standard procedures by uniformly trained personnel for measuring lung function and FeNO in study children. On the other hand, several limitations should be acknowledged. First, the cross-sectional nature of this study makes addressing the inference of causality not possible in the present study. However, the second wave of subject recruitment and data collection has been ongoing. Thus, we will be able to determine the causal relationship between BMI and lung volume and airflow in children once data are available in the near future. Second, residual confounding by other unmeasured factors remain possible, although several important variables have been taken into account in the analyses. Third, further investigation is needed to determine whether these results are generalizable to other ethnic populations.

In conclusion, findings in this study provide supportive evidence that excess weight disproportionately impacts lung volumes and airflow which is reflected by increase in FVC, FEV_1_, PEF, and FEF_25-75_, but decrease in FEV_1_/FVC ratio in a sample of children from the general population; and the results also suggest that the observed effect is independent of atopic status. Excess weight inversely affects FeNO in atopic but not in non-atopic children. Further investigation on underlying mechanisms is warranted.

## Supporting information

S1 Table Multivariable analysis of associations of BMI categories with lung function variables (percentage of predicted values). (DOCX)

S2 Table Multivariable analysis of associations of BMI z-scores with lung function variables (percentage of predicted values). (DOCX)

S3 Table Multivariable analysis of associations of BMI z-scores with lung function variables (percentage of predicted values), stratified by atopy. (DOCX)

S4 Table Multivariable analysis of associations of BMI z-scores with lung function variables and FeNO in subjects with and without asthma. (DOCX)
