# Supplementary material for: Obesity disproportionately impacts lung volumes, airflow and exhaled nitric oxide in children
Source: PLoS One. 2017 Apr 4;12(4):e0174691. doi: 10.1371/journal.pone.0174691 (PMC5380337; doi:10.1371/journal.pone.0174691)
Supplement: S1 Table — (DOCX) [file pone.0174691.s001.docx]

**S1 Table. Multivariable analysis of associations of BMI categories with lung function variables (percentage of predicted values)**

|  | **Total subjects (*n*=1,717)**^$^ | | **Subjects without asthma**  **(*n*=1,509)**^†^ | |
| --- | --- | --- | --- | --- |
|  | **β (95% CI)** | ***P*** | **β (95% CI)** | ***P*** |
| **FVC % predicted (%)** |  |  |  |  |
| Thinness | -0.953 (-4.92, 3.014) | 0.64 | -0.796 (-4.755, 3.162) | 0.69 |
| Normal weight | Reference | － | Reference | － |
| Overweight | 0.274 (-1.087, 1.635) | 0.69 | 0.657 (-0.79, 2.104) | 0.37 |
| Obesity | 1.887 (-0.286, 4.059) | 0.09 | 2.491 (0.136, 4.845) | **0.04** |
| **FEV_1_ % predicted (%)** |  |  |  |  |
| Thinness | -1.488 (-5.302, 2.325) | 0.44 | -1.377 (-5.15, 2.396) | 0.47 |
| Normal weight | Reference | － | Reference | － |
| Overweight | -0.427 (-1.735, 0.882) | 0.52 | -0.363 (-1.742, 1.016) | 0.61 |
| Obesity | 0.986 (-1.103, 3.074) | 0.36 | 1.689 (-0.555, 3.933) | 0.14 |
| **FEV_1_/FVC % predicted (%)** |  |  |  |  |
| Thinness | -0.154 (-2.56, 2.253) | 0.9 | -0.202 (-2.577, 2.173) | 0.87 |
| Normal weight | Reference | － | Reference | － |
| Overweight | -0.737 (-1.563, 0.088) | 0.08 | -1.055 (-1.923, -0.187) | **0.02** |
| Obesity | -0.775 (-2.093, 0.543) | 0.25 | -1.714 (-2.127, 0.698) | 0.32 |
| **PEF % predicted (%)** |  |  |  |  |
| Thinness | -3.338 (-9.521, 2.845) | 0.29 | -3.365 (-9.551, 2.821) | 0.29 |
| Normal weight | Reference | － | Reference | － |
| Overweight | -1.11 (-3.231, 1.011) | 0.31 | -1.88 (-4.14, 0.381) | 0.10 |
| Obesity | 1.405 (-1.981, 4.791) | 0.42 | 1.312 (-2.367, 4.991) | 0.48 |
| **FEF_25-75_ % predicted (%)** |  |  |  |  |
| Thinness | -4.265 (-11.307, 2.778) | 0.24 | -4.278 (-11.289, 2.733) | 0.23 |
| Normal weight | Reference | － | Reference | － |
| Overweight | -0.361 (-2.777, 2.055) | 0.77 | -1 (-3.562, 1.563) | 0.44 |
| Obesity | 0.335 (-3.521, 4.192) | 0.87 | 1.032 (-3.138, 5.201) | 0.63 |

BMI: body mass index; CI: confidence interval; FVC: forced vital capacity; FEV_1_: forced expiratory volume in 1 second; PEF: peak expiratory flow; FEF_25-75_: forced expiratory flow at 25-75%; ppb, parts per billion.

^*^BMI categories were determined by using a set of age and sex specific BMI cut-off values for ages 2-18 years that were constructed to pass the adult BMI cut-offs at age 18 for thinness (<17 kg/m^2^), normal weight (≧17 and <25 kg/m^2^), overweight (≧25 and <30 kg/m^2^) and obesity (≧30 kg/m^2^). *P* values less than 0.05 are in bold.

^$^Among 1,717 study subjects acceptable lung function and FeNO measurements were obtained in 1,672 and 1,651 subjects, respectively. Adjusted for age, sex, asthma and active smoking.

^†^208 subjects with asthma (n=179) or missing data (n=29) were excluded from the analyses. Adjusted for age, sex, and active smoking.
