# Supplementary material for: Obesity disproportionately impacts lung volumes, airflow and exhaled nitric oxide in children
Source: PLoS One. 2017 Apr 4;12(4):e0174691. doi: 10.1371/journal.pone.0174691 (PMC5380337; doi:10.1371/journal.pone.0174691)
Supplement: S2 Table — (DOCX) [file pone.0174691.s002.docx]

**S2 Table. Multivariable analysis of associations of BMI z-scores with lung function variables (percentage of predicted values)^*^**

|  | **Total subjects (*n*=1,717)**^$^ | | **Subjects without asthma (*n*=1,509)**^†^ | |
| --- | --- | --- | --- | --- |
|  | **β (95% CI)** | ***P*** | **β (95% CI)** | ***P*** |
| **FVC % predicted (%)** | 1.428 (0.955, 1.901) | **<0.001** | 1.644 (1.146, 2.143) | **<0.001** |
| **FEV_1_ % predicted (%)** | 1.078 (0.621, 1.535) | **<0.001** | 1.287 (0.81, 1.764) | **<0.001** |
| **FEV_1_/FVC % predicted (%)** | -0.374 (-0.664, -0.084) | **0.01** | -0.381 (-0.684, -0.079) | **0.01** |
| **PEF % predicted (%)** | 0.971 (0.227, 1.716) | **0.01** | 0.82 (0.032, 1.609) | **0.04** |
| **FEF_25-75_ % predicted (%)** | 0.908 (0.059, 1.756) | **0.04** | 1.087 (0.194, 1.98) | **0.02** |

BMI: body mass index; CI: confidence interval; FVC: forced vital capacity; FEV_1_: forced expiratory volume in 1 second; PEF: peak expiratory flow; FEF_25-75_: forced expiratory flow at 25-75%; ppb, parts per billion.

^*^BMI z-score was treated as a continuous variable. *P* values less than 0.05 are in bold.

^$^Among 1,717 study subjects acceptable lung function and FeNO measurements were obtained in 1,672 and 1,651 subjects, respectively. Adjusted for age, sex, asthma and smoking.

^†^208 subjects with asthma (n=179) or missing data (n=29) were excluded from the analyses. Adjusted for age, sex, and smoking.
