# Supplementary material for: Obesity disproportionately impacts lung volumes, airflow and exhaled nitric oxide in children
Source: PLoS One. 2017 Apr 4;12(4):e0174691. doi: 10.1371/journal.pone.0174691 (PMC5380337; doi:10.1371/journal.pone.0174691)
Supplement: S3 Table — (DOCX) [file pone.0174691.s003.docx]

**S3 Table. Multivariable analysis of associations of BMI z-scores with lung function variables (percentage of predicted values), stratified by atopy^*^**

|  | **Atopy (*n*=757)**^$^ | | **No Atopy (*n*=564)**^$^ | |
| --- | --- | --- | --- | --- |
|  | **β (95% CI)** | ***P*** | **β (95% CI)** | ***P*** |
| **FVC % predicted (%)** | 1.115 (0.377, 1.853) | **0.003** | 1.7 (0.822, 2.577) | **<0.001** |
| **FEV_1_ % predicted (%)** | 0.886 (0.183, 1.59) | **0.01** | 1.233 (0.428, 2.039) | **0.003** |
| **FEV_1_/FVC % predicted (%)** | -0.268 (-0.717, 0.181) | 0.24 | -0.399 (-0.893, 0.095) | 0.11 |
| **PEF % predicted (%)** | 0.936 (-0.162, 2.035) | 0.10 | 0.837 (-0.548, 2.222) | 0.24 |
| **FEF_25-75_ % predicted (%)** | 1.059 (-0.222, 2.34) | 0.11 | 0.834 (-0.659, 2.326) | 0.27 |
|  | **IgE >=100 kU/L (*n*=615)** | | **IgE< 100 kU/L (*n*=706)** | |
| **FVC % predicted (%)** | 1.104 (0.301, 1.908) | **0.007** | 1.608 (0.815, 2.401) | **<0.001** |
| **FEV_1_ % predicted (%)** | 0.866 (0.098, 1.634) | **0.03** | 1.207 (0.476, 1.937) | **0.001** |
| **FEV_1_/FVC % predicted (%)** | -0.319 (-0.817, 0.18) | 0.21 | -0.325 (-0.771, 0.121) | 0.15 |
| **PEF % predicted (%)** | 0.821 (-0.413, 2.054) | 0.19 | 1.012 (-0.2, 2.224) | 0.10 |
| **FEF_25-75_ % predicted (%)** | 0.822 (-0.596, 2.24) | 0.26 | 1.153 (-0.181, 2.487) | 0.09 |
|  | **IgE >= 77.7 kU/L (*n*=696)** | | **IgE < 77.7 kU/L (*n*=625)** | |
| **FVC % predicted (%)** | 1.428 (0.649, 2.207) | **<0.001** | 1.263 (0.442, 2.084) | **0.003** |
| **FEV_1_ % predicted (%)** | 1.128 (0.4, 1.856) | **0.002** | 0.928 (0.155, 1.7) | **0.02** |
| **FEV_1_/FVC % predicted (%)** | -0.363 (-0.829, 0.104) | 0.13 | -0.251 (-0.726, 0.223) | 0.30 |
| **PEF % predicted (%)** | 0.796 (-0.367, 1.959) | 0.18 | 1.032 (-0.26, 2.324) | 0.12 |
| **FEF_25-75_ % predicted (%)** | 1.025 (-0.305, 2.354) | 0.13 | 0.954 (-0.471, 2.379) | 0.19 |

BMI: body mass index; CI: confidence interval; FVC: forced vital capacity; FEV_1_: forced expiratory volume in 1 second; PEF: peak expiratory flow; FEF_25-75_: forced expiratory flow at 25-75%; ppb, parts per billion.

^*^BMI z-score was treated as a continuous variable. Atopy was defined as a positive Phadiatop Infant test (≥0.35 PAU/L). We also used total serum IgE to define atopy at a customary cutoff of 100 kU/L and a previously reported optimal cutoff of 77.7 kU/L [19] as a sensitivity test. Adjusted for age, sex, asthma, and active smoking. *P* values less than 0.05 are in bold.

^$^Atopy data was available in 1,321 of 1,717 subjects who provided blood samples for testing. As a result, number of subjects evaluated in Supplemental Table 4 is a subset of subjects examined in Supplemental Tables 2 and 3.
