# Supplementary material for: Obesity disproportionately impacts lung volumes, airflow and exhaled nitric oxide in children
Source: PLoS One. 2017 Apr 4;12(4):e0174691. doi: 10.1371/journal.pone.0174691 (PMC5380337; doi:10.1371/journal.pone.0174691)
Supplement: S4 Table — (DOCX) [file pone.0174691.s004.docx]

**S4 Table. Multivariable analysis of associations of BMI z-scores with lung function variables and FeNO in subjects with and without asthma^*^**

|  | **Subjects with asthma (*n*=179)** | | **Subjects without asthma (*n*=1,509)** | |
| --- | --- | --- | --- | --- |
|  | **β (95% CI)** | ***P*** | **β (95% CI)** | ***P*** |
| **FVC (L)** | 0.073 (0.031, 0.115) | **0.001** | 0.115 (0.099, 0.131) | **<0.001** |
| **FEV_1_ (L)** | 0.05 (0.01, 0.085) | **0.01** | 0.088 (0.074, 0.101) | **<0.001** |
| **FEV_1_/FVC ratio (%)** | -0.584 (-1.48, 0.313) | 0.2 | -0.606 (-0.873, -0.339) | **<0.001** |
| **PEF (L/s)** | 0.171 (0.078, 0.264) | **<0.001** | 0.135 (0.102, 0.168) | **<0.001** |
| **FEF_25-75_ (L/s)** | 0.023 (-0.047, 0.092) | 0.52 | 0.073 (0.049, 0.097) | **<0.001** |
| **Ln FeNO (ppb)** | -0.021 (-0.14, 0.098) | 0.73 | -0.04 (-0.075, -0.005) | **0.03** |

BMI: body mass index; CI: confidence interval; FVC: forced vital capacity; FEV_1_: forced expiratory volume in 1 second; PEF: peak expiratory flow; FEF_25-75_: forced expiratory flow at 25-75%; ppb, parts per billion.

^*^BMI z-score was treated as a continuous variable. Subjects with missing data on asthma (n=29) were excluded from the analyses. Adjusted for age, sex, and smoking. *P* values less than 0.05 are in bold.
